# Exploring the Dose‐Response Relationship Between Nature‐Based Outdoor Activities, Nature Connectedness and Social Health In Adolescents: A Quasi‐Experimental Controlled Study

**DOI:** 10.1002/jad.12515

**Published:** 2025-05-14

**Authors:** Eun Yeong Choe, Jen Yoohyun Lee, Shimin Zhu

**Affiliations:** ^1^ School of Design, The Hong Kong Polytechnic University Hong Kong China; ^2^ Department of Applied Social Sciences The Hong Kong Polytechnic University Hong Kong China

**Keywords:** adolescence, empathy, nature connectedness, prosocial behaviour, social connection

## Abstract

**Introduction:**

Nature‐based outdoor activities have been recognised not only as educational means to raise awareness and sensitivity to environmental challenges but also as upstream health promotion interventions for adolescents. This study investigates the relationship between nature‐based activity duration and adolescents’ nature connectedness and social health.

**Methods:**

Based on a quasi‐experimental design, 110 students (58 male and 52 female; 13–16 years) were recruited from Hong Kong secondary schools. We conducted a pre/post/follow‐up survey on nature connectedness and social health with different durations of nature‐based outdoor activities. Thirty‐two students participated in a 1‐day nature‐based outdoor activity session, 33 participated in a 4‐day nature‐based outdoor activity programme, and 45 were not assigned any activities and served as the control group.

**Results:**

The nature‐based activities significantly and immediately improved the nature connectedness of the one‐ and 4‐day groups, but this improvement continued only in the 4‐day group at the 1‐month follow‐up. Increases in social connectedness, empathy and prosocial behaviour were also observed only in the 4‐day group.

**Conclusion:**

Our findings suggest that longer‐term encounters with nature result in a greater sense of nature connectedness and more significant improvements in social health compared to a one‐off visit. Maintaining a sustained exposure–response relationship with nature can help young people have a physically and socially healthy adolescence.

## 1 Introduction

In recent years, nature‐based outdoor activities have not only been recognised as educational means of raising awareness and sensitivity to the ecosystem and environmental challenges but have also emerged as an upstream health promotion intervention for adolescents. Being out in nature can lead to physical benefits, such as increased physical activity and obesity prevention (Cleland et al. 2008), and emotional benefits, such as stress reduction and improved mood (Rowley et al. 2022). More importantly, there are emerging concerns about adolescent health problems associated with social well‐being, such as social exclusion (Redmond et al. 2024; Tomova et al. 2021) and loneliness (Twenge et al. 2021). Social health is determined by the quality of interpersonal relationships and a general sense of connectedness to others (Goosby et al. 2013). Having good‐quality social health is linked to better emotional regulation, increased self‐esteem, positive health behaviours and a lower risk of mental illness (Blum et al. 2022). McAnally et al. (2018) showed that spending time in nature positively influences adults’ social health, such as increasing their social connectedness and prosocial behaviour (i.e. actions that require some cost to the self to provide some benefit to others). As for adolescents, despite the importance of social health in their lives, far too little attention has been paid to the beneficial impacts of nature experiences on their social connectedness and prosocial behaviour (Rowley et al. 2022), and limited evidence for the relationship between prosocial behaviour and nature exposure has been obtained (e.g. Putra et al. 2021). Thus, it is crucial to explore the influence of nature‐based outdoor activities on adolescents’ social well‐being.

At the individual level, social health can be defined as the ability to interact with others and form relationships through a sense of connectedness, acceptance and belonging (Arbuthnott 2023). Prosocial behaviour – voluntary behaviour intended to benefit others (Eisenberg et al. 2006) – indicates a higher level of social health. Several attempts have been made to explore the benefits of nature exposure to social health. In a study by Piff et al. (2015), participants were instructed to spend 1 min looking up at trees or adjacent tall buildings, after which the researchers dropped a pen. The results demonstrated that those who gazed at trees were significantly more likely to help retrieve the pen than those who looked at the building. Similarly, Guéguen and Stefan (2016) conducted a glove‐dropping experiment at either the entrance to or the exit of an urban park. They found that the participants were more likely to retrieve the dropped glove after walking through the park than when entering it (i.e. after walking through the urban streets). Castelo et al. (2021) also conducted an experiment in which the participants were asked to fill out a short survey form at the entrance of a nature hiking trail either before they began their hike or after they completed it. Upon completion of the survey, the participants were asked to either enter their names into a draw for an iPad or donate money to a local charity. The participants at the exit of the nature hiking trail were significantly more likely to choose the prosocial option (i.e. making a donation) than the self‐focused option (i.e. entering their names into the draw for an iPad). Additionally, a recent review of several experimental studies (Arbuthnott 2023) concluded that exposure to nature increases prosocial decisions and social engagement, decreases antisocial behaviour and aggression and improves social connectedness.

While previous studies have found positive impacts of nature exposure on social health, in most cases, their experiments were limited to brief nature exposures and their immediate effects, and they focused on the general adult population. In this study, we applied interventions based on structured programmes to determine the effects of nature exposure on adolescent nature connectedness and social health, supplementing previously obtained evidence. We also investigated whether different durations of nature‐based outdoor activities affect social health outcomes for adolescents. To date, few studies have investigated the relationship between nature‐based activity duration and adolescents’ nature connectedness. Stern et al. (2008) explored the influences of three‐ and 5‐day residential environmental education programmes on participants’ nature connectedness, environmental stewardship, interest in learning and discovery and awareness. They found significant positive short‐term effects on all outcomes of interest, and longer programmes were associated with longer‐term retention of environmental knowledge and awareness among the participants at the 3‐month follow‐up. Similarly, Braun and Dierkes (2017) examined the effects of two nature‐based outdoor interventions that differed in duration on students’ degree of nature connectedness. The results demonstrated that both the one‐ and 5‐day interventions had an immediate positive impact on nature connectedness, while the 5‐day intervention resulted in significantly stronger, longer‐term effects. Unlike their study, ours primarily focused on participants’ nature connectedness rather than on the benefits they received from nature‐based outdoor interventions. Other studies have also shown that sustained benefits were obtained from repeated interventions that involved nature observation, mindfulness practice or engagement with nature (Choe et al. 2020). Through a longer‐term concentration on nature, situational interest can develop into individual interest, which may lead to deeper and more focused connectedness after an intervention. More recently, a meta‐analysis by Choe and Sheffield (2024) supported the idea that long‐term nature exposure is an effective way of promoting an emotional bond with nature. However, since each study employed different nature‐based programmes tailored to its specific objectives and methodologies, the findings were difficult to generalise. Much uncertainty remains about the relationship between nature‐based activity duration and adolescents’ nature connectedness and social health.

Therefore, the purpose of this study was to further investigate the nature‐connectedness and social health benefits of nature‐based outdoor activities for adolescents. A quasi‐experimental controlled study was conducted to explore the dose–response relationship between nature‐based outdoor activities and adolescents’ social health. We investigated whether longer‐term experiences with nature would result in greater nature connectedness and social health benefits (i.e. social connectedness, empathy, prosocial behaviour and self‐esteem) compared to one‐off nature‐based outdoor activities.

## 2 Methods

### 2.1 Participants

Hong Kong public secondary schools were invited to participate in nature‐based activity sessions. Once a school agreed to participate, we invited students to join either a single‐day outdoor nature‐based activity session or a 4‐day outdoor nature‐based activity programme. To minimise potential bias, we recruited students for the control group from the same school. Using G*Power version 3.1 (Faul et al. 2007) and setting a power of 0.95 with an alpha level of 0.05, it was determined that 69 participants (23 per group) would be sufficient to detect a small to medium effect size (*f*(v) = 0.20). Initially, 116 students agreed to participate in nature‐based outdoor activities. Six participants who did not complete the baseline questionnaire were excluded. Thus, 110 participants were included in the analysis (58 male and 52 female; mean age 14.42; range 13–16 years). Thirty‐two students attended a single‐day nature‐based outdoor activity session, 33 attended a 4‐day nature‐based outdoor activity programme, and 45 were not assigned any tasks and served as the control group. There were no significant differences in age (*χ* ^*2*^ = 9.09, *p* = 0.17) or gender (*χ* ^*2*^ = 4.17, *p* = 0.12) between the experimental and control group. All participants were of Chinese ethnicity (100%), and five students (4.5%) had engaged in similar nature‐based activities within the previous three months.

### 2.2 Design and Procedure

A quasi‐experimental design was used to estimate the impact of intervention duration on secondary students’ well‐being outcomes. The intervention assignment was determined by the school teachers based on the school schedule. The experimental procedure was explained to potential participants in a recruitment letter, which required them to send their informed consent before participating. After consenting to participate in the study, all students were asked to complete a questionnaire comprising five psychometrically validated scales to measure their baseline social health and well‐being. Completion of the questionnaire took approximately 10–15 mins. Once the participants had completed the questionnaire, one group was asked to attend a 1‐day nature‐based outdoor activity session, and another group was asked to attend a 4‐day nature‐based outdoor activity programme. The activities, which took place in an urban park, consisted of walking/hiking, sensory exploration and mindfulness practice. They aimed to enhance the students’ nature connectedness and were designed for a 1‐day nature‐based outdoor session (a total of 4–5 h) and were repeated four times over a 4‐day programme (a total of 19 h) at the same site. The control group was not assigned any tasks. After the experiment, all the participants were asked to complete two follow‐up questionnaires identical to the one they had filled out before the experiment (T1): one immediately after the completion of their programme (T2) and another one month later (T3). The experiment was conducted from May to July 2024. This study was approved by the PolyU Institutional Review Board under the Research Committee (Reference Number: HSEARS20241122002).

### 2.3 Measurements

#### 2.3.1 Inclusion of Nature In Self Scale

The Inclusion of Nature in Self (INS; Schultz 2001) scale measures the degree to which individuals feel interconnected with nature. This single‐item 7‐point pictorial scale presents a series of progressively more interconnected circles representing the ‘self’ and ‘nature’. Scores range from 1 to 7, with the least overlapping circle receiving a score of 1 (complete separation from nature) and the most overlapping circle receiving a score of 7 (complete connection to nature). The INS scale has high internal consistency, with a Cronbach's alpha of 0.91.

#### 2.3.2 Social Connectedness Scale

The Social Connectedness Scale ‐ Revised (SCS‐R; Lee et al. 2001; Lee and Robbins 1998) measures respondents’ degree of interpersonal closeness and degree of difficulty in sustaining a sense of closeness in their social world. The SCS‐R contains 20 items (10 positive and 10 negative) measured on a 6‐point Likert scale ranging from 1 (strongly disagree) to 6 (strongly agree). Negatively worded items (e.g. ‘I feel disconnected from the world around me’) are reverse‐scored and summed with the positive items (e.g. ‘I feel comfortable in the presence of strangers’) to obtain a total score. The scores range from 20 to 120, with higher scores indicating a greater sense of belonging, closeness and support. The SCS‐R's Cronbach's alpha is 0.82, which indicates a high level of internal consistency.

#### 2.3.3 Brief Interpersonal Reactivity Index

The Brief Interpersonal Reactivity Index (B‐IRI; Ingoglia et al. 2016) measures the degree to which respondents can empathise with others. The B‐IRI contains 16 items consisting of four sub‐scales. Among the four sub‐scales, empathic concern (e.g. ‘I would describe myself as a pretty soft‐hearted person’) and perspective‐taking (e.g. ‘I try to look at everybody's side in a disagreement before I make a decision’) were used to obtain a total IRI score (Wang et al. 2020). The eight items are measured on a 5‐point Likert scale ranging from 0 (describes me very well) to 4 (does not describe me well). Scores range from 0 to 32, with higher scores indicating a greater sense of empathy. The B‐IRI's Cronbach's alpha is 0.79, indicating a high level of internal consistency.

#### 2.3.4 Strengths and Difficulties Questionnaire

The Strengths and Difficulties Questionnaire (SDQ; Goodman 1997) measures the strengths and difficulties in respondents’ behavioural traits. The SDQ comprises five sub‐scales designed to measure emotional symptoms (e.g. ‘I worry a lot’), conduct problems (e.g. ‘I get very angry and often lose my temper’), hyperactivity (e.g. ‘I am constantly fidgeting or squirming’), peer relationship problems (e.g. ‘Other children or young people pick on me or bully me’) and prosocial behaviour (e.g. ‘I am kind to younger children’), each consisting of five items. A total of 25 items are measured on a 3‐point Likert scale ranging from 0 (not true) to 2 (certainly true). In this study, we used a three‐subscale division of the SDQ: internalising problems (emotional + peer relationship problems, 10 items), externalising problems (conduct + hyperactivity symptoms, 10 items) and the prosocial scale (5 items) (Goodman et al. 2010). For each subscale, higher scores indicate greater degrees of difficulty and strength. The SDQ has high internal consistency, with a Cronbach's alpha of 0.87 for internalising problems, 0.89 for externalising problems and 0.92 for prosocial behaviour.

#### 2.3.5 The Rosenberg Self‐Esteem Scale

The Rosenberg Self‐Esteem Scale (RSES; Rosenberg and Rosenberg 1965) measures respondents’ self‐esteem. The RSES comprises 10 items (5 negative and 5 positive), which are measured on a 4‐point Likert scale ranging from 1 (strongly disagree) to 4 (strongly agree). Negatively worded items (e.g. ‘I feel that I do not have much to be proud of’) are reverse‐scored and summed with the positively worded items (e.g. ‘I feel that I have a number of good qualities’) to obtain a total score. Scores range from 10 to 40, with higher scores indicating higher levels of self‐esteem. The RSES's Cronbach's alpha is 0.89, indicating a high level of internal consistency.

### 2.4 Analysis Strategy

All the analyses were conducted using SPSS for Windows version 24.0, with an alpha of 0.05. An *η* ^*2*^ below 0.01 was considered a small effect, between 0.01 and 0.06 a medium effect and above 0.14 a large effect (Cohen 1988). First, χ^2^ tests and analysis of variance (ANOVA) were used to examine the differences at baseline. Next, multivariate analysis of variance (MANOVA) was used to investigate the effects of the intervention on the three groups at three time points. These analyses incorporated a between‐subjects factor (1‐day, 4‐day and control) and three time points of interventions (T1, T2 and T3) for social health outcomes. If there was a significant interaction, follow‐up analysis was performed using one‐way ANOVAs to compare groups, and where a group was significant, this was explored using post hoc comparisons with Tukey's HSD. Paired samples t‐tests were also used to further investigate the differences within each group.

## 3 Results

Univariate ANOVA revealed no baseline differences in any of the study measures by environment (*p* > 0.05). Next, MANOVA was used to determine the main effects of time and environment on all the measures. An initial MANOVA that included gender revealed no gender interactions in the multivariate or univariate effects (*p* > 0.05). However, it revealed that the main effect of time was significant (*F*(14, 94) = 1.81, *p* = 0.04, *η* ^*2*^ = 0.21), indicating that the measures changed over time. There were also significant interactions between the three study groups (the 1‐day, 4‐day and control groups) and the three time points (T1, T2 and T3) (*F*(28,186) = 2.22, *p* < 0.001, *η* ^*2*^ = 0.252) at the multivariate level, indicating that the groups differed in terms of changes in social health outcomes over time. Table 1 shows the means, SD and 95% CI for all the measurements by environment at the three time points.

**Table 1:** Means, standard deviations and 95% confidence intervals for the measurements at three time points.

		T1		T2		T3	
	*n*	*M* (SD)	95% CI	*M* (SD)	95% CI	*M* (SD)	95% CI
**INS**							
One‐day group	32	3.472 (1.666)	2.873, 4.073	3.752 (1.374)	3.108, 4.142	3.591 (1.523)	3.047, 4.142
Four‐day group	33	3.362 (1.598)	2.798, 3.934	3.910 (1.681)	3.313, 4.511	4.092 (1.673)	3.501, 4.682
Control (reference)	45	3.331 (1.371)	2.922, 3.742	3.222 (1.062)	2.903, 3.542	3.271 (0.942)	2.984, 3.552
**SCS‐R**							
One‐day group		68.912 (8.158)	65.973, 71.851	69.253 (10.182)	65.582, 72.921	66.134 (10.482)	63.881, 70.802
Four‐day group		71.002 (7.532)	68.332, 73.668	73.973 (10.781)	70.151, 77.793	76.026 (10.281)	72.382, 79.677
Control (reference)		70.313 (8.379)	67.564, 73.071	72.491 (5.373)	70.570, 73.523	71.272 (5.011)	69.502, 72.279
**B‐IRI**							
One‐day group	32	17.791 (5.423)	15.873, 19.712	18.393 (4.851)	16.682, 20.112	18.302 (4.625)	16.663, 19.942
Four‐day group	33	17.972 (4.452)	16.362, 19.573	19.061 (3.812)	17.690, 20.443	19.912 (3.210)	18.751, 21.064
Control (reference)	45	18.134 (5.033)	16.623, 19.643	19.113 (4.234)	17.845, 20.381	19.113 (4.158)	17.862, 20.364
**SDQ (internalising problems)**							
One‐day group	32	8.656 (3.671)	7.332, 9.981	8.534 (2.022)	7.802, 9.262	8.314 (2.221)	7.511, 9.113
Four‐day group	33	8.703 (2.602)	7.771, 9.623	8.613 (2.103)	7.861, 9.353	7.762 (1.710)	7.152, 8.372
Control (reference)	45	8.798 (1.972)	8.210, 9.391	8.798 (2.032)	8.192, 9.412	8.912 (1.878)	8.352, 9.481
**SDQ (externalising problems)**							
One‐day group	32	7.631 (1.432)	7.112, 8.142	7.751 (2.272)	6.931, 8.572	7.782 (2.502)	6.883, 8.681
Four‐day group	33	7.552 (1.481)	7.024, 8.071	7.523 (1.890)	6.852, 8.192	7.356 (1.598)	6.802, 7.934
Control (reference)	45	7.532 (2.283)	6.851, 8.223	7.672 (2.134)	7.033, 8.311	7.532 (2.045)	6.923, 8.152
**SDQ (prosocial behaviour)**							
One‐day group	32	4.841 (2.202)	4.045, 5.642	4.912 (1.889)	4.221, 5.589	5.062 (2.124)	4.304, 5.832
Four‐day group	33	5.302 (1.103)	4.912, 5.689	6.091 (1.892)	5.423, 6.762	7.523 (2.711)	6.556, 8.473
Control (reference)	45	5.331 (1.897)	4.762, 5.902	4.962 (1.645)	4.463, 5.453	5.002 (1.623)	4.511, 5.492
**RSES**							
One‐day group	32	25.031 (3.287)	23.852, 26.221	25.061 (4.643)	23.392, 26.743	25.163 (5.962)	23.013, 27.302
Four‐day group	33	25.182 (3.832)	23.822, 26.543	25.452 (2.824)	24.463, 26.452	25.362 (3.081)	24.272, 26.461
Control (reference)	45	25.292 (3.628)	24.203, 26.384	25.823 (2.001)	25.222, 26.421	25.996 (2.001)	25.403, 26.602

### 3.1 Nature Connectedness (INS)

The analysis showed both a significant main effect for time (*F*(2, 106) = 3.823, *p* = 0.022, *η* ^*2*^ = 0.028) and a significant interaction effect between time and group (*F*(4, 214) = 2.134, *p* = 0.026, *η* ^*2*^ = 0.056). An ANOVA revealed no differences between the groups at T1 (*F*(2, 107) = 0.078, *p* = 0.931, *η* ^*2*^ = 0.012) or T2 (*F*(2, 107) = 2.432, *p* = 0.089, *η* ^*2*^ = 0.043). However, the differences in the groups’ mean INS scores were significant at T3 (*F*(2, 107) = 3.482, *p* = 0.029, *η* ^*2*^ = 0.052). The post hoc test indicated that the mean score of the 4‐day group (*M* = 4.092, SD = 1.673) was significantly different from that of the control group (*M* = 3.271, SD = 0.942). The mean score of the 1‐day group did not differ significantly from that of the other groups at T3.

**Figure 1:**
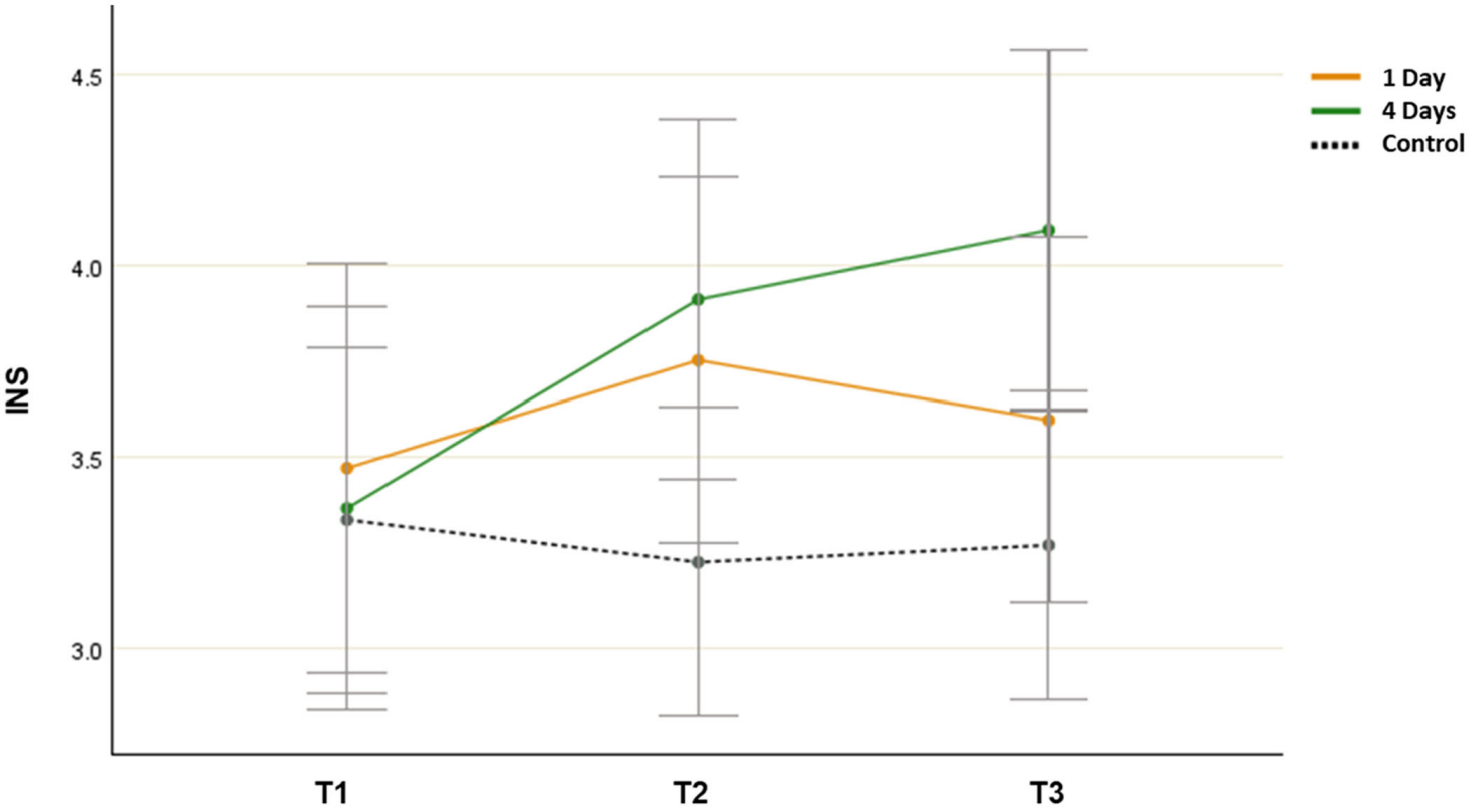
Changes in mean INS scores (nature connectedness; error bars denote a 95% confidence interval).

Paired samples t‐tests revealed that there was a statistically significant increase in mean INS scores from T1 (*M* = 3.362, SD = 1.598) to T3 (*M* = 4.092, SD = 1.673; *t*(32) = − 2.596, *p* = 0.014, *η* ^*2*^ = 0.452) in the 4‐day group. However, a significant improvement was observed in the 1‐day group from T1 (*M* = 3.472, SD = 1.666) to T2 (*M* = 3.752, SD = 1.374; *t*(31) = − 1.612, *p* = 0.051, *η* ^*2*^ = 0.986) but not from T1 to T3 (*M* = 3.591, SD = 1.523; *t*(32) = − 0.551, *p* = 0.298, *η* ^*2*^ = 0.102). Thus, the participants’ nature connectedness was immediately improved in both nature‐based outdoor activity groups; however, the effect only persisted at follow‐up (T3) in the 4‐day group (Figure 1).

### 3.2 Social Connectedness (SCS‐R)

A repeated measures ANOVA revealed a significant group‐by‐time interaction effect (*F*(4, 214) = 3.001, *p* = 0.034, *η* ^*2*^ = 0.048). The ANOVA revealed no differences between the groups at T1 (*F*(2,107) = 0.532, *p* = 0.592, *η* ^*2*^ = 0.102) or T2 (*F*(2,107) = 2.452, *p* = 0.088, *η* ^*2*^ = 0.098). However, the differences between the groups’ mean SCS‐R scores were significant at T3 (*F*(2,107) = 9.278, *p* < 0.001, *η* ^*2*^ = 0.153). The post hoc test indicated that the mean score of the 4‐day group (*M* = 76.026, SD = 10.281) was significantly different from the mean scores of the 1‐day group (*M* = 66.134, SD = 10.482) and the control group (*M* = 71.272, SD = 5.011). The mean score of the 1‐day group did not differ significantly from that of the control group at T3.

**Figure 2:**
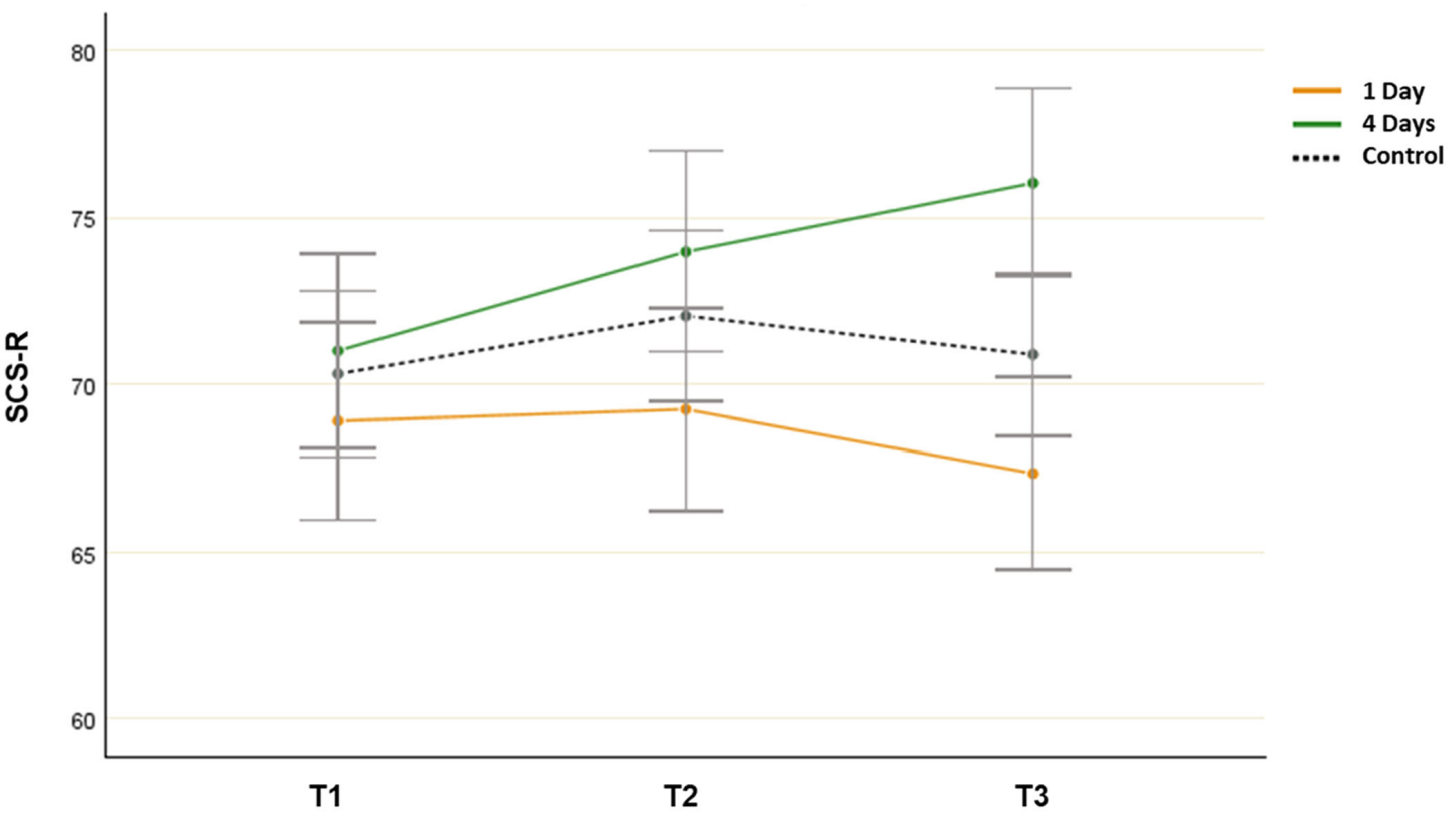
Changes in mean SCS‐R scores (social connectedness; error bars denote a 95% confidence interval).

Paired samples t‐tests revealed that there was a statistically significant decrease in the mean SCS‐R score from T1 (*M* = 71.002, SD = 7.532) to T3 (*M* = 76.026, SD = 10.281; *t*(31) = − 2.942, *p* = 0.003, Cohen's *d* = 0.512) in the 4‐day group. However, no significant improvement was found from T1 (*M* = 68.912, SD = 8.158) to T3 (*M* = 66.134, SD = 10.482; *t*(32) = 1.092, *p* = 0.143, Cohen's *d* = 0.193) in the 1‐day group or from T1 (*M* = 70.313, SD = 8.379) to T3 (*M* = 71.272, SD = 5.011; *t*(44) = − 0.423, *p* = 0.343, Cohen's *d* = 0.103) in the control group. Thus, the participants’ social connectedness improved only in the 4‐day group (Figure 2).

### 3.3 Empathy (B‐IRI)

The analysis showed a significant main effect for time (*F*(2, 106) = 3.742, *p* = 0.027, *η* ^2^ = 0.068), but there was no significant group‐by‐time interaction effect (*F*(4, 214) = 0.653, *p* = 0.629, *η* ^*2*^ = 0.012).

**Figure 3:**
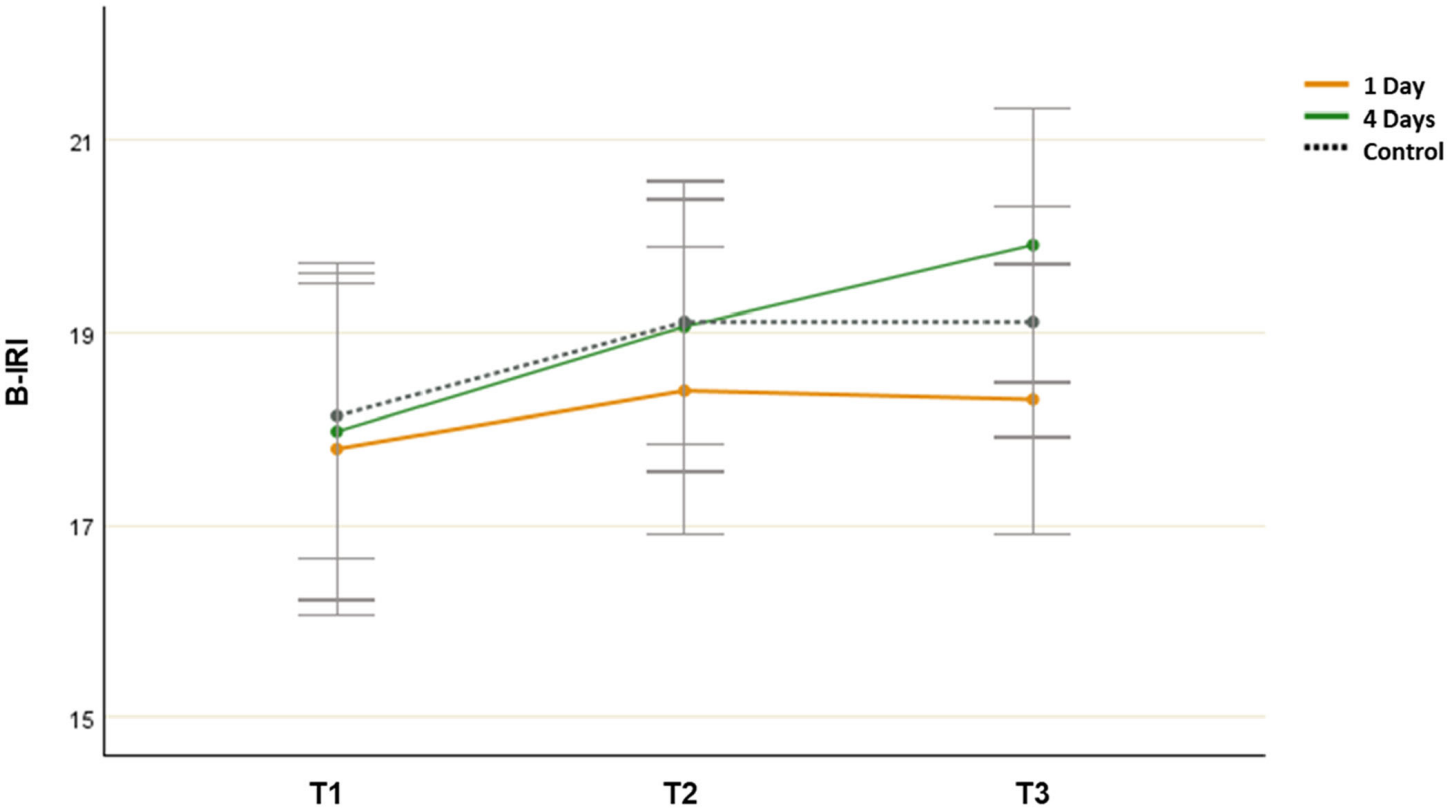
Changes in mean B‐IRI scores (empathy; error bars denote a 95% confidence interval).

Paired samples t‐tests revealed that there was a statistically significant decrease in the mean B‐IRI score from T1 (*M* = 17.971, SD = 4.452) to T3 (*M* = 19.912, SD = 3.210; *t*(31) = −3.332, *p* = 0.001, Cohen's *d* = 0.381) in the 4‐day group. However, no significant improvement was found from T1 (*M* = 17.791, SD = 5.423) to T3 (*M* = 18.302, SD = 4.625; *t*(32) = −0.571, *p* = 0.287, Cohen's *d* = 0.090) in the 1‐day group and from T1 (*M* = 18.134, SD = 5.033) to T3 (*M* = 19.113, SD = 4.158; *t*(44) = −1.415, *p* = 0.164, Cohen's *d* = 0.208) in the control group. Thus, the participants’ levels of empathy improved only in the 4‐day group (Figure 3).

### 3.4 Internalising Problems (SDQ)

A repeated measures ANOVA showed no significant main effect for time (*F*(2, 106) = 0.497, *p* = 0.613, *η* ^*2*^ = 0.013) and no significant group‐by‐time interaction effect (*F*(4, 214) = 0.172, *p* = 0.947, *η* ^*2*^ = 0.028). The ANOVA revealed no differences between the groups at T1 (*F*(2,107) = 0.031, *p* = 0.966, *η* ^*2*^ = 0.01) or T2 (*F*(2,107) = 0.182, *p* = 0.843, *η* ^*2*^ = 0.013). However, there were significant differences in internalising problems between the groups at T3 (*F*(2,107) = 3.402, *p* = 0.042, *η* ^*2*^ = 0.056). The post hoc test indicated that the mean score of the 4‐day group (*M* = 7.762, SD = 1.710) was significantly different from that of the control group (*M* = 8.912, SD = 1.878). The mean score of the 1‐day group did not differ significantly from that of the other groups at T3.

**Figure 4:**
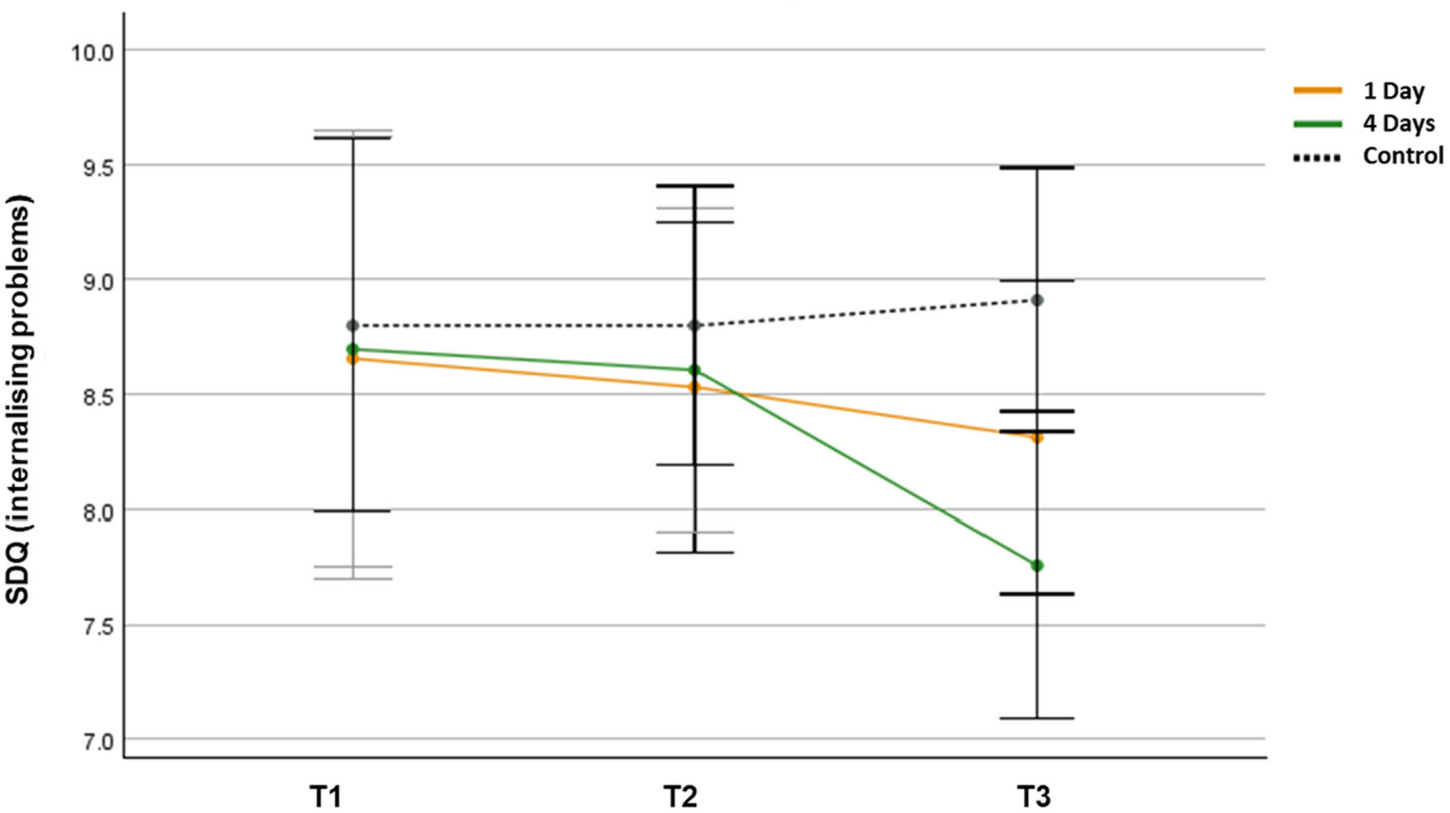
Changes in mean SDQ scores (internalising problems; error bars denote a 95% confidence interval).

Paired samples t‐tests revealed that there was a statistically significant decrease in internalising problems from T1 (*M* = 8.703, SD = 2.602) to T3 (*M* = 7.762, SD = 1.710; *t*(31) = 2.232, *p* = 0.023, *η* ^*2*^ = 0.397) in the 4‐day group. However, no significant improvement was found from T1 (*M* = 8.656, SD = 3.671) to T3 (*M* = 8.314, SD = 2.221; *t*(32) = 0.423, *p* = 0.342, *η* ^*2*^ = 0.068) in the 1‐day group and from T1 (*M* = 8.798, SD = 1.972) to T3 (*M* = 8.912, *SD* = 1.878; *t*(44) = − 0.451, *p* = 0.335, *η* ^*2*^ = 0.072) in the control group. Thus, the participants’ internalising problems were reduced only in the 4‐day group (Figure 4).

### 3.5 Externalising Problems (SDQ)

**Figure 5:**
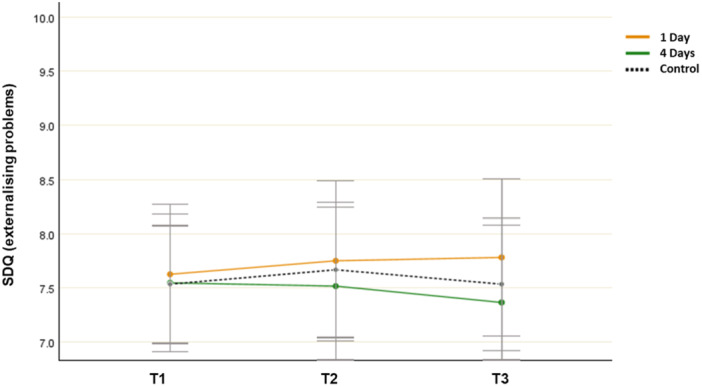
Changes in mean SDQ scores (externalising problems; error bars denote a 95% confidence interval).

A repeated measures ANOVA showed no significant main effect for time (*F*(2, 106) = 0.153, *p* = 0.862, *η* ^*2*^ = 0.858) and no significant group‐by‐time interaction effect (*F*(4, 214) = 0.174, *p* = 0.953, *η* ^*2*^ = 0.014). The ANOVA revealed no differences between environments at each time point (*p* > 0.05). Although further analysis revealed a decreased mean SDQ score from T1 to T3 (7.552 and 7.356, respectively) in the 4‐day group, this finding was not statistically significant (*p* = 0.399) (Figure 5).

### 3.6 Prosocial Behaviour (SDQ)

The analysis showed both a significant main effect for time (*F*(2, 106) = 8.982, *p* < 0.001, *η* ^*2*^ = 0.078) and a significant group‐by‐time interaction effect (*F*(4, 214) = 10.192, *p* < 0.001, *η* ^*2*^ = 0.162). The ANOVA revealed no differences between environments at T1 (*F*(2, 107) = 0.801, *p* = 0.452, *η* ^*2*^ = 0.020). However, the differences between the groups’ mean SDQ scores were significant at T2 (*F*(2, 107) = 4.793, *p* = 0.012, *η* ^*2*^ = 0.081) and T3 (*F*(2, 107) = 15.632, *p* < 0.001, *η* ^*2*^ = 0.201). The post hoc test results indicated that the mean score of the 4‐day group (*M* = 6.091, SD = 1.892) was significantly different from those of the 1‐day group (*M* = 4.912, SD = 1.889) and the control group (*M* = 4.962, SD = 1.645) at T2. At T3, the mean score of the 4‐day group (*M* = 7.523, SD = 2.711) was significantly different from that of the control group (*M* = 5.002, SD = 1.623). The mean score of the 1‐day group did not differ significantly from that of the other groups at T3.

**Figure 6:**
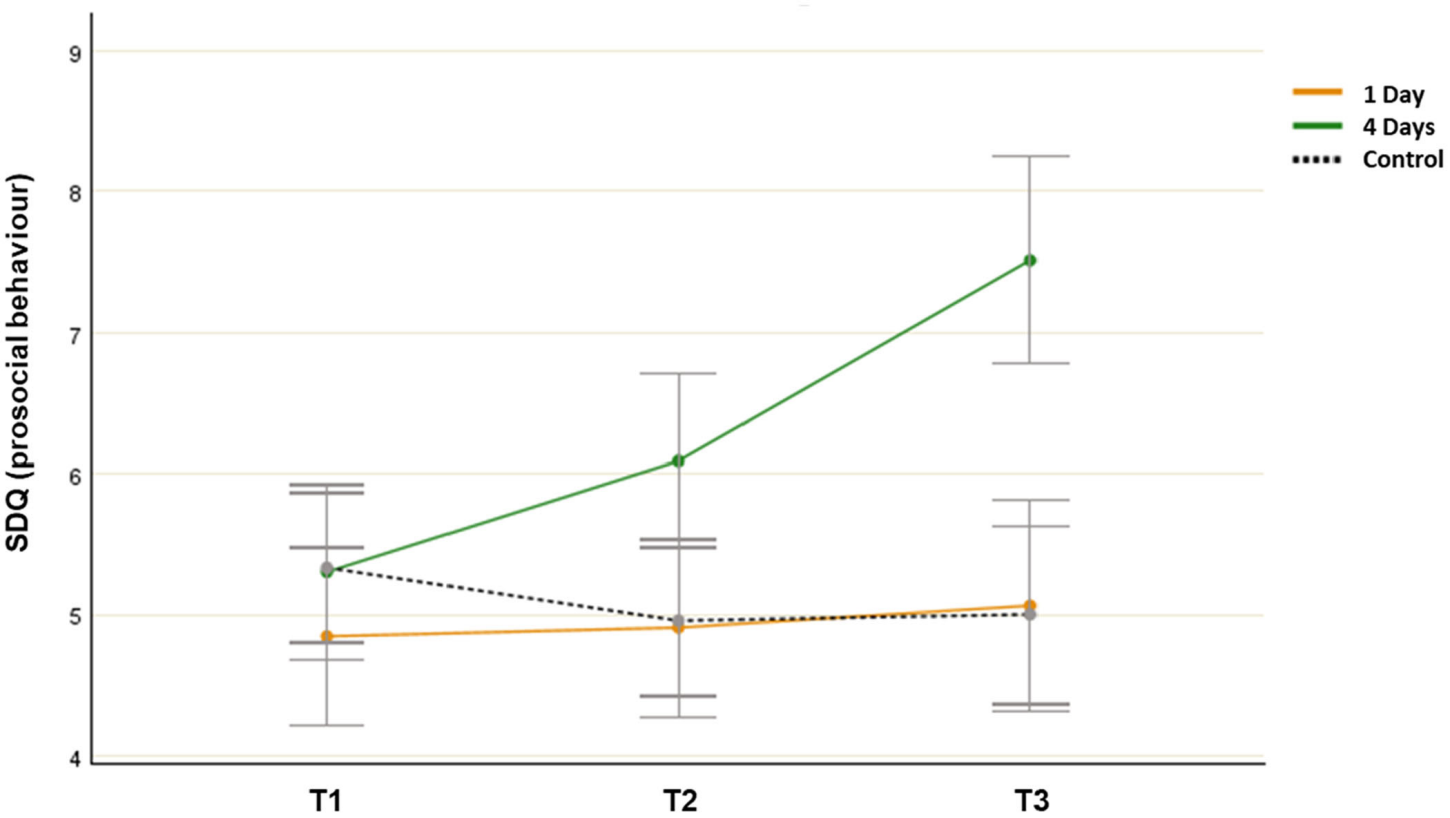
Changes in mean SDQ scores (prosocial behaviour; error bars denote a 95% confidence interval).

Paired samples t‐tests revealed that there was a statistically significant increase in SDQ scores from T1 (*M* = 5.302, SD = 1.103) to T3 (*M* = 7.523, SD = 2.711; *t*(31) = −5.398, *p* < 0.001, *η* ^*2*^ = 0.942) in the 4‐day group. However, no significant improvement was found from T1 (M = 4.841, SD = 2.202) to T3 (*M* = 5.062, SD = 2.124; *t*(32) = − 0.432, *p* = 0.328, *η* ^*2*^ = 0.067) in the 1‐day group or from T1 (*M* = 5.331, SD = 1.897) to T3 (*M* = 5.002, SD = 1.623; *t*(44) = 2.192, *p* = 0.024, *η* ^*2*^ = 0.331) in the control group. Thus, the participants’ prosocial behaviour was improved only in the 4‐day group (Figure 6).

### 3.7 Self‐Esteem (RSES)

**Figure 7:**
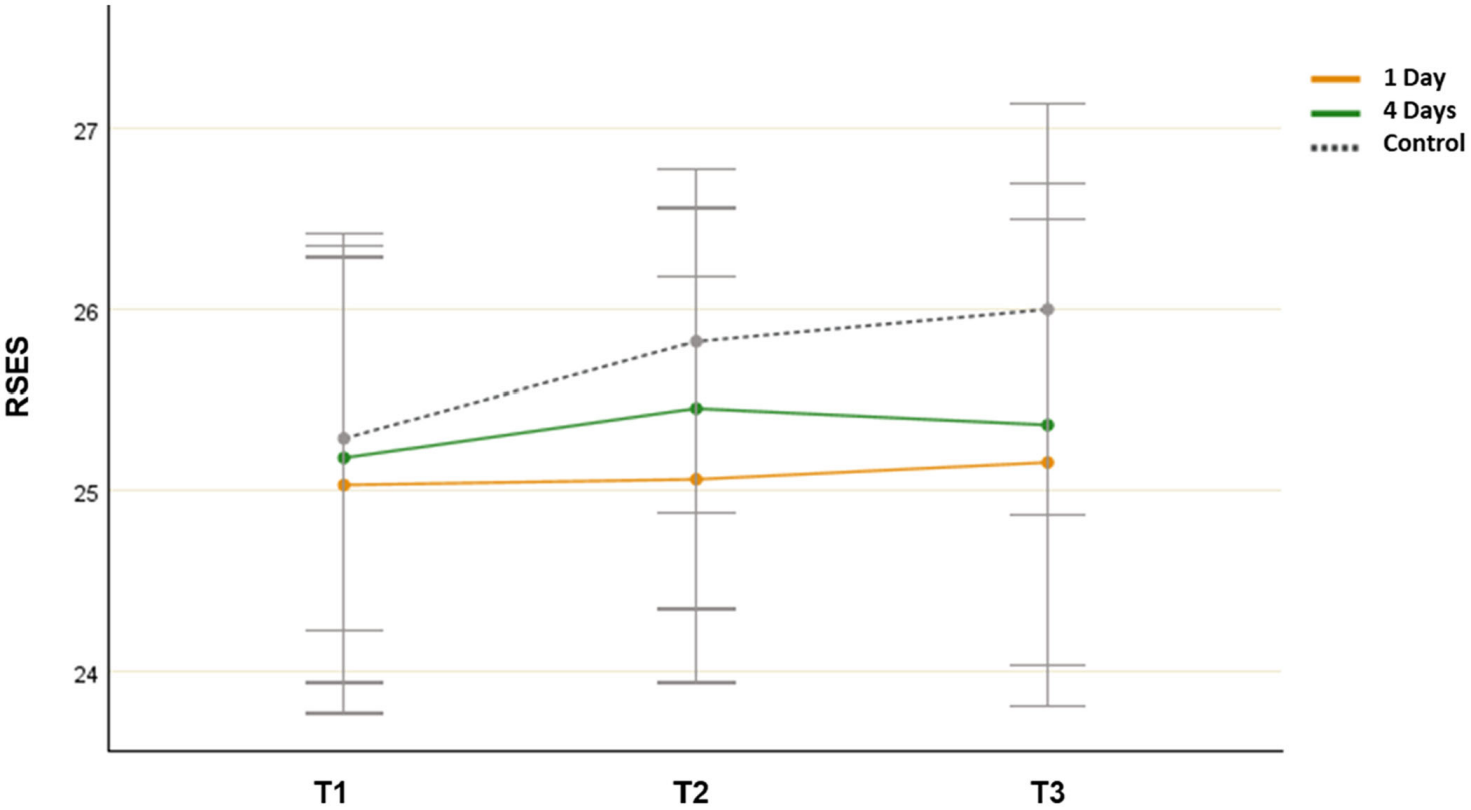
Changes in mean RSES scores (self‐esteem; error bars denote a 95% confidence interval).

A repeated measures ANOVA showed no significant main effect for time (*F*(2, 106) = 0.501, *p* = 0.612, *η* ^*2*^ = 0.013) and no significant group‐by‐time interaction effect (*F*(4, 214) = 0.173, *p* = 0.954, *η* ^*2*^ = 0.032). The ANOVA revealed no differences between environments at each time point (*p* > 0.05). Although further analysis revealed an increased mean RSES score from T1 to T3 (25.182 and 25.362, respectively) in the 4‐day group, this finding was not statistically significant (*p* = 0.399) (Figure 7).

## 4 Discussion

This study investigated the relationship between nature‐based activity duration and adolescents’ nature connectedness and social health outcomes. We found that nature‐based activities generally improved participants’ nature connectedness and social health. Nevertheless, significant improvements in social health were observed only in the 4‐day group. Notably, participants’ sense of nature connectedness continued to increase in the 4‐day group at the 1‐month follow‐up but not in the 1‐day group. Longer‐term experiences with nature evidently result in greater nature connectedness and improvements in social health compared to a one‐off visit.

First, we found that nature‐based outdoor activities improved the participants’ connectedness to nature. Both the one‐ and 4‐day groups showed immediate improvements after the interventions. However, this improvement in participants’ nature connectedness continued for 1 month in the 4‐day group but not in the 1‐day group. This result is consistent with the results of previous studies (e.g., Braun and Dierkes 2017; Stern et al. 2008), indicating that longer‐term exposure to nature is more effective in promoting adolescents’ emotional bonds with nature. Through a longer‐term concentration on nature, situational interest can develop into individual interest, which potentially leads to deeper and more focused connectedness after the intervention.

Second, we found a significant improvement in the participants’ social connectedness in the 4‐day group but not in the 1‐day group. This accords with the finding of Oh et al. (2022) that people who visited gardens more frequently were more likely to have a strong sense of belonging, connectedness and inclusion within their local community. Several studies have explored the direct impacts of nature exposure on social interactions, such as using green schoolyards to build supportive social groups (Chawla et al. 2014). Resonating with these studies, our results support the idea that longer nature exploration sessions have more sustained effects on participants’ social connectedness than one‐off visits. Indeed, we found sustained improvement even one month after the completion of the intervention in the 4‐day group but not in the 1‐day group. This suggests that programmes encouraging adolescents to regularly engage in various nature‐based outdoor activities could increase their sense of nature connectedness and their associated perception of social connectedness in the long term.

Our study also showed a significant improvement in empathy and prosocial behaviour in the 4‐day group but not in the 1‐day group. While previous studies (Castelo et al. 2021; Piff et al. 2015) have shown that brief nature exposure, such as looking at trees or taking a short walk in a park, enhances empathy and prosocial behaviour, our findings suggest that this does not apply to adolescents. Prosocial behaviour stems from empathy, an emotional response that arises from understanding or perceiving another's emotional state or condition (Acar and Torquati 2015), and such empathy towards other living things can be developed from interactions with nature (Cheng and Monroe 2012). However, many other factors influence the development of empathic attitudes and prosocial behaviour throughout adolescence, including individual characteristics, environmental influences and experiences and interactions with family and friends (Silke et al. 2018). In our study, one factor that could have influenced the participants’ development of prosocial behaviour was peer interaction during the prolonged activity, which provided opportunities for helping, cooperating and sharing among themselves. Thus, adolescents’ empathy and prosocial behaviour towards nature and people can be developed not only through longer‐term programmes but also through well‐planned and well‐implemented content in nature‐based activities. Fostering adolescents’ understanding and respect for life through programmes centred on active interactions can lay the foundation for prosocial behaviour towards nature and other human beings.

In both the one‐ and 4‐day groups, although the participants’ internalising problems improved after the interventions, their externalising problems did not. Internalising problems include worry, anxiety, depression and social withdrawal (Goodman et al. 2010). This finding broadly supports the findings of other studies on the mental health benefits of nature, demonstrating the positive impact of nature exposure on adolescents’ mood, stress, anxiety, depression and hedonic tone (Bowen et al. 2016; Li et al. 2018). By contrast, externalising problems include hyperactivity, impulsivity, aggression and rule violation (Goodman et al. 2010). Our findings contradict those of previous studies indicating that nature exposure positively influences anger and aggressive behaviour. A study by Kuo and Sullivan (2001) showed that residents living in relatively barren buildings exhibit more aggression and violence than their counterparts living in buildings surrounded by greenery. Similarly, forest‐related environmental education resulted in decreased aggression, irritability, restlessness, emotional instability and negativism in adolescents diagnosed with affective and behavioural disorders (Macháčková et al. 2021).

Finally, we observed no significant improvement in participants’ self‐esteem in any of the groups. Previous studies on the relationship between outdoor activities and adolescents’ self‐esteem have been somewhat inconsistent (Mygind et al. 2019; Tillmann et al. 2018). Hayhurst et al. (2015) and Hunter et al. (2013) asserted that interactions with friends and others in outdoor environments are essential to adolescents’ social lives and can contribute to the development of their self‐esteem. However, Barton et al. (2015) and Wood et al. (2014) found that nature exposure has no additive effect on adolescents’ and children's self‐esteem compared to exposure to other environments. These inconsistencies suggest that further research in this area is needed. Future research could investigate how different types of nature interactions impact adolescents’ self‐esteem.

This study has some limitations. First, a non‐randomised study design could be considered a weakness. Since the assignment was not random, it was challenging to account for potential confounding variables in the results. Further experiments using random assignment could reduce selection bias and confounding variables and provide more definitive evidence that can subsequently improve nature‐based interventions for adolescents. Second, we did not examine the effects of individual socioeconomic status or previous experiences with similar outdoor activities. Further research controlling for individual differences would help establish a greater degree of accuracy in this matter. Third, this study did not account for whether the participants engaged in additional nature visits or nature‐based outdoor activities, which may have affected the outcomes of the interventions. These results therefore, need to be interpreted with caution. Lastly, we did not measure the participants’ individual levels of engagement with nature during the intervention. [Author(s)] confirmed that the quality of adolescents’ engagement with nature is a significant predictor of their nature connectedness; thus, overlooking the individual participants’ nature engagement levels may have been a shortcoming of this study. Actively engaging with nature by noticing, appreciating or experiencing it through the senses leads to a stronger connection than passive engagement. An increased focus on the measurement of not only the quantity but also the quality of interventions would help achieve greater accuracy in the dose–response relationship between nature‐based outdoor activities and social health among adolescents.

Notwithstanding these limitations, the present study has several implications for adolescents in the education and health sectors. Our results indicate that longer nature exposure increases adolescents’ sense of nature connectedness, which in turn improves their social well‐being. The evidence supporting increased nature connectedness through long‐term interventions suggests that it is essential to design routine or repeated nature exposure activities to foster both nature connectedness and social opportunities. For instance, creating accessible gardens on school campuses could optimise the provision of regular nature exposure. Health practitioners might consider implementing ‘nature‐based social prescriptions’ (Menhas et al. 2024) that encourage adolescents experiencing social isolation or loneliness to engage in nature‐based outdoor activities or volunteer in local green spaces, as these settings are likely to facilitate meaningful social interactions.

## 5 Conclusion

This study shed some light on the importance of nature‐based outdoor activity duration by assessing the social health benefits of nature engagement for adolescents. Given the foundational significance of social relationships with friends and others for adolescents’ health and well‐being, examining how social health can be improved through nature‐based outdoor experiences provides valuable insights into refining education and health programmes for teens. Our findings demonstrate that longer‐term encounters with nature result in a greater sense of nature connectedness and improved social health compared to a one‐off visit. Thus, maintaining a longer‐term exposure–response relationship with nature can help young people have a physically and socially healthy adolescence. It is essential for educational organisations and policymakers to focus their efforts on providing more opportunities for longer‐term engagement with nature through regular outdoor activities or gardening programmes for adolescents.

## Data Availability

The data that support the findings of this study are available on request from the corresponding author. The data are not publicly available due to privacy or ethical restrictions.
